# Interprofessional and Inter‐Organisational Collaboration in the COVID‐19 Vaccination Programme: Lessons From North Central London

**DOI:** 10.1111/jan.16775

**Published:** 2025-01-26

**Authors:** Helen T. Allan, Sophia Drakopoulou, Miranda Willis, Michael Traynor, Deborah Scott, Fiona Suthers, Karen Colfer, Dan Levene

**Affiliations:** ^1^ Faculty of Health, Social Care and Education Middlesex University London UK; ^2^ Faculty of the Arts and Creative Industries Middlesex University London UK; ^3^ North Central London Secondary Care Covid‐19 Vaccination Lead UCLH NHS Foundation Trust London UK; ^4^ North Central London Clinical Programme Lead for Covid‐19 Vaccination UCLH NHS Foundation Trust London UK; ^5^ Department of Nursing & Midwifery Faculty of Health, Social Care & Education, Middlesex University London UK; ^6^ UCLH NHS Foundation Trust London UK; ^7^ NCL ICB London UK

**Keywords:** COVID‐19 vaccination programme, inter‐organisational collaboration, interprofessional collaboration

## Abstract

**Aim:**

To discuss inter‐organisational collaboration in the context of the successful COVID‐19 vaccination programme in North Central London (NCL).

**Design:**

An action research study in 2023–2024.

**Methods:**

Six action research cycles used mixed qualitative methods.

**Results:**

Four findings are presented which illustrate inter‐organisational collaboration across professional and organisational boundaries: working in the action research group, learning to work as an action research group, working collaboratively in new ways, working outside professional, occupational and organisational silos. These themes are discussed in relation to the literature on interprofessional and inter‐organisational collaboration.

**Conclusion:**

The COVID‐19 vaccination programme offered a way out of the pandemic. Between December 2020 and February 2022, 2.8 M people were vaccinated by the NCL Vaccination team in an example of inter‐organisational collaboration between science, health and community. Staff on the vaccination programme worked inter‐organisationally in new ways to achieve this. In NCL several thousand local residents joined the NHS to work with healthcare professionals including nurses, nursing associates and students to deliver the programme in new ways which are illustrative of inter‐organisational collaboration.

**No Patient or Public Contribution:**

No PPI within this study.

**Implications for the Profession and/or Patient Care:**

The implications for the profession and for healthcare organisations of the findings are that, in contrast to traditional ways of working which have been entrenched in silos of professional knowledge and expertise, health professionals are able to work in new ways and find inter‐organisational work satisfying. This has implications for patients as it has the potential to improve communication between very different organisations and as the vaccination programme shows, results in successful public health vaccination rates.

**Impact:**

This study set out to create a public resource for learning (for future pandemics or other works of national effort) to commemorate the collaborative efforts of the diverse vaccination workforce and volunteers involved in the programme. Participation in the COVID‐19 vaccination programme had a profound effect on NHS clinical and professional staff, on partners across business and volunteer organisation in North Central London and on volunteers from the public in North Central London. Inter‐organisation collaboration has been sustained after the delivery of the vaccination programme in North Central London; innovative ways of working have been introduced in the local community to deliver ongoing vaccinations and wider prevention activities and the partnership between academia and clinical practice. The research findings have had an impact on the research participants and the wider public through the website created as a public resource to commemorate the COVID‐19 vaccination programme in North Central London.

**Reporting Method:**

The consolidated criteria for reporting qualitative studies (COREQ) was used as a guide throughout data collection and analysis.

**Patient or Public Contribution:**

The public were involved as participants in this study. They did not participate in the study design.

**Summary**

- What does this paper contribute to the wider global clinical community?
  ○ In NCL more than half the population lives in the 40% most deprived areas nationally. NCL is also ethnically diverse. Barnet and Camden have larger Asian communities, making up nearly 20% of the population in those boroughs. Haringey and Enfield have a similar proportion of Black residents. Across NCL, 20% of residents come from non‐British White backgrounds. As a result of interprofessional and inter‐organisational working, despite NCL being the second most deprived Integrated Care System in London with an ethnically diverse population, it had Covid‐19 vaccination rates above the London average.
  ○ In contrast to traditional ways of working which have been entrenched in silos of professional knowledge and expertise, health professionals were able to work in new ways across organisations with patient benefits in vaccination delivery. Working together to ensure that stakeholders are joined up and are providing services that are accessible and inclusive has potential benefits in wider healthcare activities in the future – particularly in reducing health inequalities.

## 1 Introduction

The COVID pandemic forced healthcare professionals (HCPs) to work differently and to collaborate in new ways (Ashcroft et al. 2023; Yorke et al. 2022). In ‘*new approaches to delivering care, [based on] a greater understanding of the skill, value and flexibility of the whole health and care workforce’* (Care Quality Commission 2020). The COVID‐19 vaccination programme, which peaked during the summer of 2021, required collaboration and innovation to set up new sites and deliver at pace. It was an unprecedented programme of work undertaken during a period of national and international instability. By February 2022, nearly 2.8 m doses had been provided to individuals in London by vaccinators in North Central London (NCL). Members of the NCL Vaccination programme included volunteers, administrators, vaccinator trainers from Middlesex University, NHS staff who were co‐opted from their pre‐pandemic roles as HCPs, health service managers as well as collaborators working in social care, businesses, local councils and voluntary organisations within the NCL footprint.

In this paper, we argue that working in the NHS during the vaccination programme in NCL included inter‐organisational collaboration: interprofessional working with non‐HCPs working in NHS trusts (procurement, construction, workforce, communications, property and finance), NHSE (National Health Service England, the executive body of the British Government Department of Health and Social Care which oversees the commissioning side of the NHS nationally), the local integrated care boards (which commission NHS services for local populations) and inter‐organisational working with volunteers, voluntary agencies, local councils, local businesses and local universities. Similarly, amongst the researchers who included partners from NCL, a strong and effective inter‐organisational collaboration was formed, leveraging the existing relationships between NCL members that had developed during the COVID‐19 pandemic.

## 2 Background

As Green and Johnson (2015) argue, collaboration involves putting the community or client first, the organisation second, oneself last, that is, rethinking how professions and other partners work together differently. Inter‐organisational collaboration is the term mostly used in business to describe collaboration across and between organisations (Green and Johnson 2015). Collaboration is a mutually beneficial and well‐defined relationship entered into by two or more organisations to achieve common goals (Mattessich and Monsey 1992, 7). Businesses have used collaboration for many years to share costs, spread risk and reduce supply chain uncertainty whilst forming strategic economic alliances that also serve as fertile grounds for innovation and learning (Vangen and Huxham 2003; Powell, Koput, and Smith‐Doerr 1996).

Interprofessional collaboration (IPC) requires practitioners to work together across disciplines and institutional boundaries; to value each other's differences and work to incorporate each discipline in the shared, integrated work (Allan et al. 2005; Schot, Tummers, and Noordegraaf 2020). Interprofessional collaboration involves an integrated approach to patient care across different health disciplines working together well to construct interdependency (Leathard 2003; D'Amour et al. 2005; Reeves et al. 2016). The relationship includes a commitment to a definition of mutual relationships and goals, a jointly developed structure and shared responsibility, mutual authority and accountability for success and the sharing of resources and rewards (Mattessich and Monsey 1992, 7).

IPC amongst researchers can help build informational networks, encourage different ways of thinking, and stimulate new solutions to old problems (Green and Johnson 2015). Trust is an important factor for collaboration in research (Green and Johnson 2015) but successful IPC is as much down to the individual professional as the social and organisational context in which they work (Schot, Tummers, and Noordegraaf 2020). This theoretical framing would include an exploration of actors' values, beliefs and intentions towards their professional practice (Thomson and Perry, 2006; Allan et al. 2023). In a systematic review of IPC, Schot, Tummers, and Noordegraaf (2020) found that HCPs achieved IPC in three ways: by bridging professional, social, physical and task‐related gaps, by negotiating overlaps in roles and tasks, and by creating spaces to communicate about interprofessional collaboration.

There is broad, general agreement (Littlechild and Smith 2013) that, to meet the future health needs of societies, the education of the workforce has to be less professionally isolated and divisive (Green and Johnson 2015). There is good evidence that IPC has been successfully introduced into acute and primary care (Schot, Tummers, and Noordegraaf 2020). However, it is shown that at the ‘grassroots’ this change is difficult to bring about and evaluate (Barr et al. 2000; Elston and Holloway 2001; Hudson 2002). Across different studies, the 1st author has found that staff recognise the need for such change; they adopt IPC but organisational structures (Allan et al. 2014), organisational and HCP's cultural beliefs (Allan and Barber 2005; Allan et al. 2014), attitudes to professional identity and roles (Allan and Barber 2005; Allan and Evans 2022) and ways of thinking (Allan and Evans 2022; Allan et al. 2023) make IPC challenging. Such challenges to IPC are similar to what Schickler (2004) described as ‘professional tribalism’ where knowledge is organised in a uni‐professional or tribalist fashion. In such a framework, there is little flexibility to share ideas and knowledge or to work together. Smith (2004) argues that tribalism is based on professionals’ tendency to draw on ‘knowledge silos’. The metaphor of silos is useful in understanding how knowledge is created within disciplines and communicated within those same disciplines but necessarily externally to those professions. To work collaboratively becomes a challenge to this silo mentality because working in this way requires shared access to ‘knowledge, transparency and exposure to new ideas, methodologies and different approaches to practice’ (Smith 2004, 116).

IPC during the COVID‐19 pandemic was essential for not only patient but staff safety (Yorke, Smith, and Mostrom 2022). Yorke, Smith, and Mostrom (2022) argue that pre‐pandemic, whilst NHS staff worked interprofessionally, the pandemic brought confusion and negotiation about roles and responsibilities within the healthcare team as ways of working changed rapidly. Yorke et al. emphasise that safety (risks of exposure) was a key concern as staff took on new roles. They describe how in collaborating in new conditions, new teams were formed and staff struggled ‘trying to find out who's going to be the leader’ (2022, 94). New teams working in new units across health disciplines were set up to meet the challenges of the pandemic (Hales et al. 2020; Tannenbaum et al. 2021). These challenges included limitations in the availability of personal protective equipment (PPE), managing the unknown along with the rapid pace of change, and the lack of scientific evidence to guide the treatment of patients with the disease (Butler et al. 2020). Many of these challenges confronted the HCPs and other professionals who designed the vaccination programme.

## 3 The Study

Our study aims were as follows

1. To create a public audiovisual, electronic/interactive artefact to remember the collaborative efforts of the diverse vaccination workforce and volunteers involved in the programme.
2. To analyse the ways of working which enabled the successful vaccination programme delivery.

The work was launched as ‘*Behind the Screens in the Vaccination Centre*’ in November 2023 at the Business Design Centre in Islington, a former mass vaccination centre. (See: https://storiesbehindthescreens.net).

## 4 Methodology

Action research facilitated diverse approaches to data collection and allowed the researchers to develop a comprehensive and inclusive narrative (Odelius et al. 2015). An action research group (ARG) was set up involving members of the research team at Middlesex University in the Faculties of Arts & Creative Industries and Health, Social Care and Education, and the NCL vaccination team who had designed and delivered the vaccination programme. The role of the ARG, which is unique to AR methodology, allowed for creative synergy among the ARG members who included HCPs, NHS managers, university staff who represented organisations and professions employed on the NCL vaccination programme. They represented two academic disciplines as well as five NHS workforce groups. The ARG worked inter‐organisationally and their reflections added to the data collection and analysis.

### 4.1 Methods

**FIGURE 1:**
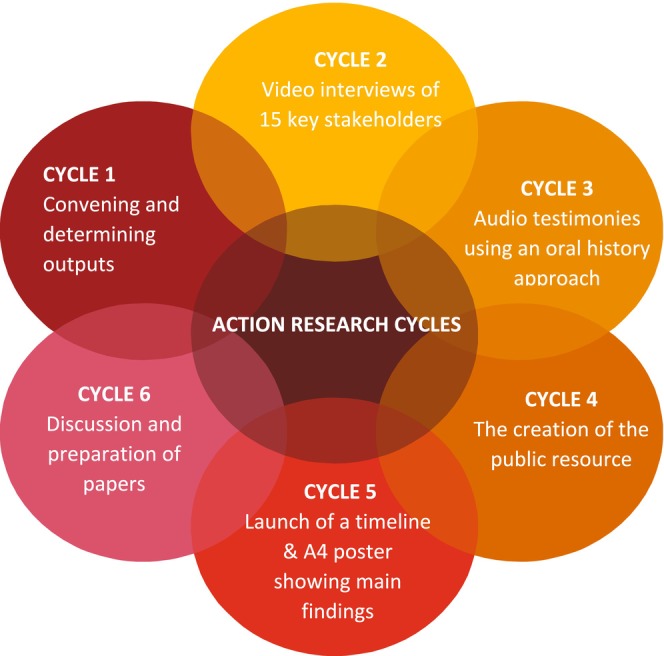
Action research cycles.

Following Odelius et al. (2015), the research involved action research cycles (Figure 1), each with separate although linked data collection and analysis. The methods included ARG minutes, video and group interviews, written and oral testimonies as well as artefacts (photographs).

The ARG held frequent online meetings to determine the project's methodology and outputs. The ARG format comprised open‐ended discussions which were recorded for minutes, whilst some included an agenda; analysis of the minutes using thematic analysis added to the study data. In the ARG, our research team from Middlesex University acted as facilitators, refraining from imposing our narrative but instead listening, compiling stories and selecting the most appropriate format to convey these stories and experiences.

Cycle 1: Convening and audio recording meetings of the ARG to discuss and determine the project's outputs. NCL members of the ARG were asked to highlight key moments of the programme in order for us to understand the duration and scope of the vaccination programme. To conceptualise the interactive artefact, the ARG reviewed various projects employing timelines. The ARG favoured design approaches utilising timelines and graphic animations. At mutually agreed points, decisions were taken for Cycle 2.

Cycle 2: To delve deeper into the intricate details of setting up and running a mass‐scale vaccination programme, we conducted seven recorded interviews with 15 key stakeholders/informants, including NCL ARG members, an NHS operational lead, an NHS communications manager, an NHS head of finance and contracts, director of NHS estates, vaccination trainer, NHS workforce coordinator. These interviews were about their experiences with the vaccination programme. One of the research team (and ARG member) conducted the interviews and the video recording was undertaken by students from the Faculty of Arts and Creative Industries. Thematic analysis of video and audio recordings was completed by two researchers (and ARG members) using an approach adapted from Sanders et al. (2023). Themes were derived inductively initially and then reanalysed using a theoretical framework developed from the literature review on inter‐organisational working. Through our ARG discussions and analysis of video interviews, we distilled key moments of the vaccination programme, such as the opening and closing of vaccination sites, community outreach projects and different phases of the programme. Based on these, we constructed a calendar‐based timeline with key events. Decisions were taken for action in Cycle 3.

Cycles 3: To capture the personal experiences of programme participants, we collected pictures, personal photos and audio testimonies using an oral history approach. Programme participants were invited via vaccination primary and secondary care email distribution lists, to record a memory and reflection of their involvement in the NCL vaccination programme using a smartphone and send in contributions via email. Over 126 self‐selecting individuals sent in contributions in the form of audio testimonies and photographs. Over 161 contributions were received, released under a creative licensing scheme granting contributors ownership of their recordings. Some individuals chose to contribute just one audio file only, some just one photograph only and some submitted both an audio file and one photograph. These 126 individuals are categorised into professional groupings in the ARG Cycle 3 in Table 2. Ownership of their testimonies was granted to professionals and volunteers, following an oral history approach. Thematic analysis of audio and video testimonies, and photos posted submitted to the researchers. Decisions were taken for Cycle 4.

Cycle 4: The creation of the public resource. At this stage, we employed a web developer to work with Dr. Sophia Drakopoulou, our design lead from Arts and & Creative Industries. This is primarily a timeline with interactive links to audio clips and photos from Cycles 2 and 3 data collection. To create the public resources a website was designed by our ARG member in the creative industries and a developer was employed to build it. The website consists of an interactive timeline with key events of the vaccination programme. Users can access content chronologically by clicking on key events on the interactive timeline we designed, or non‐linearly by clicking on floating dots and opening audio clips testimonies and photos.

**FIGURE 2:**
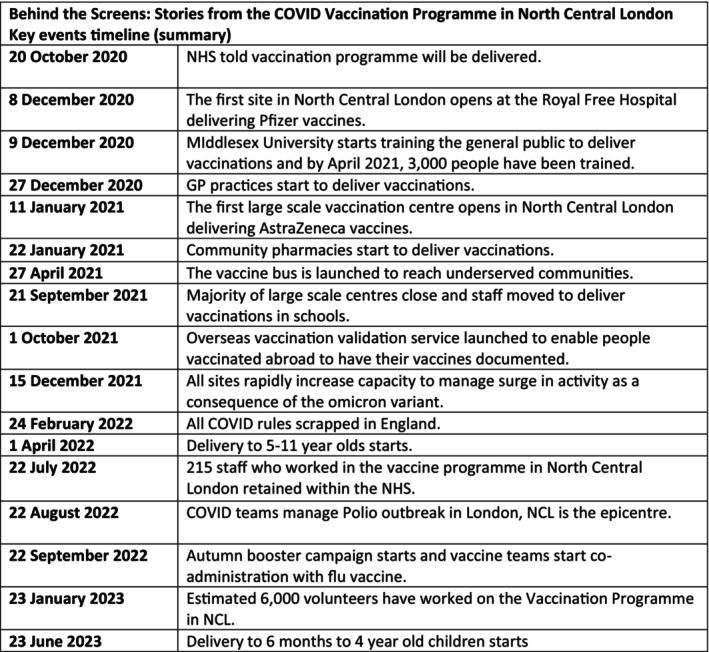
Key timeline events. The ARG identified 48 key timeline events in the programme and displayed on the website. Fifteen key timeline events are displayed here as a summary.

Cycle 5: A launch of the project with a timeline A4 poster showing the main research findings (see Figure 2). On Friday 3rd of November 2023, a press release was published and a project launch event was held at the Islington Business Design Centre (see Figure 2). Participants of the NCL Vaccination programme attended and were able to interact with the artefact and view and listen to their contributions.

Cycle 6: Discussion and preparation of papers from the research. Monitoring of website visits, dissemination via social media.

### 4.2 Ethical Considerations

After an ethical review at Middlesex University (Arts & Creative Industries Ethics Committee in July 2022 and January 2023. Reference number: 21632), recruitment for Cycle 2 was directly through ARG member and snowballing from ARG members' contacts and knowledge of key stakeholders/informants involved in the design and delivery of the vaccination programme. Consent was obtained prior to each interview in Cycle 2. Recruitment for Cycle 3 was through the NCL vaccination programme database of volunteers. The ARG members and researchers had no direct contact with the Cycle 3 participants. Implied consent was given in Cycle 3 as contributors were invited by email from the NCL vaccination programme to send in their contributions and could opt out if they wished.

### 4.3 The Website

The aim of the website was to be a repository of people's experiences and stories of the NCL COVID Vaccination Programme, with an easy‐to‐use and inclusive interface design.

The website's primary purpose is to celebrate the collaborative efforts of over 11,000 staff and volunteers who administered 2.8 million COVID‐19 vaccine doses across the five NCL boroughs (Nov 2023). The video interviews, photos and audio testimonies collected serve as historical documentation of unprecedented events, contributing to our collective understanding of how the NCL COVID Vaccination programme was established and run as a collaborative inter‐organisational programme. The website celebrates people uniting in adversity to successfully deliver a mass vaccination programme.

### 4.4 Website Content

To capture and preserve the personal experiences of the people involved, interviews with key stakeholders were conducted in Cycle 2 and testimonies of experiences beyond the managerial level of the NHS were gathered in Cycle 3. As described above, a call‐out was made to programme participants (professionals and volunteers) to share images of the moments captured during the programme as well as audio clip recordings.

These photos and audio files highlight moments of connection, solidarity, and community strength, showcasing the initiative as an extraordinary collective effort. Ownership of their recordings was shared with professionals and volunteers, following an oral history approach (Dougherty and Simpson 2012), where content is owned by the content contributors.

The inclusion of professionals' and volunteers' own words and images created a multi‐dimensional narrative, allowing users to randomly select content or follow a linear narrative by clicking on key events in the timeline. The audio testimonies, interviews with key stakeholders and images provide a comprehensive view of the challenges, achievements, and community‐centric aspects of the NHS NCL COVID Vaccination Programme. The audio testimonies emphasise local engagement, the employment of local individuals and reaching hard‐to‐reach groups, which contributed to addressing health inequalities during the pandemic and possibly beyond.

### 4.5 Timeline

Through the methods employed during the ARG meetings, as described above, the ARG constructed a timeline of key events in the vaccination programme, spanning from 2020 to 2023. The ARG created and co‐edited a collaborative document online, capturing these key timeline events, which was then shared with the website developer, creating a seamless IPC production workflow amongst a multidisciplinary working group. In total, 48 key events were identified and displayed on the website. For the design of the promotional poster and the launch event at the Islington Design Centre on 3 November 2023, these were condensed to 15 key events and presented here. See Figure 2.

### 4.6 Timeline and Content on Design and Impact

The website's multimedia storytelling approach and thematic organisation offer valuable personal accounts of how the project was managed and run. The website design uses intuitive navigation, enabling users to access key events either chronologically or non‐linearly. This original approach effectively communicates complex health‐related information to the public. The audio clips, interviews with key stakeholders and images provide a comprehensive view of the challenges, achievements and community focus of the NHS Central North London Covid Vaccination Programme. As noted in the ARG thematic analysis of the content contributed, emphasis is placed on local engagement, employing local individuals and reaching hard‐to‐reach groups, to combat and address health inequalities. All the data can be accessed via the website which serves as a collaborative storytelling project and historical record of people's memories and experiences of the vaccination programme.

## 5 Findings – Inter‐Organisational Collaboration in Producing the Artefact

Following Odelius et al. (2015), our first key finding is a process finding, that is, the ways of working which produced the artefact itself illustrate inter‐organisational collaboration in innovative ways. The ARG was interdisciplinary, drawing on expertise from various industry sectors and professions in six action research cycles (see Figure 1 and Table 2).

Through thematic analysis of the content (photos, audio testimonies, interviews and meeting transcripts), seven themes emerged (Table 1).

**TABLE 1:** Themes arising from data analysis in cycles 2 and 3.

	Themes	Descriptor
1	Size/scope of project	Huge scale of work to be delivered within a short timeframe; scale of achievement; level of public support; level of hard work; passion and baptism of fire.
2	Different types of involvement	Collaboration is a strong narrative; positive comments on working in new ways with new teams; welcome of new people into existing teams; volunteers, managers and clinical staff being engaged in a shared purpose.
3	Local working	Employing local people for local work; engaging people new to the NHS to work in NHS roles; linked to reaching hard‐to‐reach groups (health inequalities); changing lives and engaging community leaders.
4	Safety	Clinical safety paramount; tension between control; transparency and getting things done in an emergency.
5	New ways of working	Decision making was outside usual NHS ways of working; governance at the clinical level was high but finance pushed to the side with a caveat about VFM.
6	Personal journeys	Made sense of careers, validated them as people/professionals (clinical and non‐clinical including volunteers); made sense of fear of pandemic/virus; doing something; new careers, as many volunteers now have careers at the NHS.
7	Political context	Political pressure to deliver; the sense of the macro (politics) driving the action and individual.

Through the analysis of images, audio testimonies, interviews with key stakeholders and the ARG meeting transcripts, seven themes emerged that encapsulate strategies employed, challenges faced and lessons learned in implementing a mass‐scale vaccination programme within a specific timeframe (see Timeline).

On the website, content was tagged according to these themes, allowing users to filter content according to the identified themes offering a more focused exploration of specific aspects of the Covid Vaccination Programme experiences.

The seven themes that emerged from this research illustrate the unprecedented challenges faced by professionals and volunteers in setting up and running a mass vaccination programme, their personal journeys, safety concerns and outreach to the local community. However, most significantly, these themes highlight the new ways of working that emerged through IPC/or interprofessional working/practices developed during the running of the programme and continued in the ARG meetings.

## 6 Findings – Inter‐Organisational Collaboration in Participants' Stories

We now present three themes that illustrate inter‐organisational collaboration across professional and organisational boundaries (Green and Johnson 2015): learning to work as an action research group, working collaboratively in new ways, working outside professional, occupational and organisational silos.

Our argument in this paper is that the inter‐organisational collaboration in the COVID‐19 vaccination programme, and especially amongst the programme team, was the basis of our inter‐organisational collaboration in ARG.

Many of the participants in Cycles 2 and 3 described the vaccination programme as ‘*way out of the pandemic’*. Participants contrasted the hope of the vaccine with the devastation of the pandemic. Many described their involvement with both as a ‘*once in a lifetime’* experience. The vaccination programme gave voice to the exceptional effort the vaccination programme entailed. It required ‘*all hands on deck’*; ‘*really quick roll out’*; it happened ‘*at speed and succeeded’*. One university nurse trainer described setting up the training programme for the vaccinators as:

As though a train was approaching and we were laying the train tracks as we could see the train over our shoulders. (Nurse trainer NHS/Cycle 2)

### 6.1 Theme. 1 – Learning to Work as an Action Research Group

During the pandemic and in the early days of the vaccine, the mass vaccination centres, the stewards, the nurses, were in the public eye. After discussions in ARG, we realised that the work undertaken by the new teams in the vaccination programme was novel and inter‐organisational, much of it unseen by the public. We chose the title of the website ‘Behind the screens in a vaccination centre’ to reflect this hidden, unseen work:

In the background there's so much more. (ARG member/Nurse, Cycle 2)

The ARG brought together individuals who had largely worked in areas of the vaccination programme that had not been in the public eye such as the NCL secondary care COVID‐19 vaccination lead, workforce managers, trainers, finance leads and the communication team. We worked collaboratively in new ways building on strengths from our different organisations and different worldviews for over 2 years and 6 months.

I've worked with more diverse team than I ever have done…delightful, amazing to see how stable it's been despite [us] being completely different. (NHS/ARG member, Cycle 2)

The ways of working we developed built on each members' experiences such as cross‐faculty academic work, and in the case of our NHS colleagues, working with volunteer, local councils and business organisations during the pandemic which in itself demonstrates inter‐organisational collaboration.

The ARG brought together individuals from different professions and organisations to create a public resource which records the work undertaken in North Central London in the delivery of the Covid‐19 vaccination programme. (NHS ARG member, Cycle 2)

Each ARG member brought their unique insights and professional competencies to the table, fostering a collective effort in conceptualising the project. It was clear that it was not merely the professional experience and training which shaped how the ARG worked together but how personal relationships were established outside organisational structures to form a trust for actions within the ARG:

For me personally, the key challenge working as an ARG was the tension between NHS colleagues (who are used to taking and implementing decisions at pace and then ‘moving on’ to the next challenge) versus the more academic approach (where ideas are considered, reflected on, modified and re‐visited multiple times). I had to adjust to a different style of working and trust that the academic process and expertise of colleagues working at Middlesex University would produce a high quality end product. (NHS ICB lead, Cycle 2)

This recognition of difference and respect for others' expertise was described by one health researcher ARG member as working at the borders of experience:

It was about respecting and trusting other people's judgement and areas of expertise because much of the work was on the borders of my own experience. I suppose my concrete contribution was doing all those interviews that were filmed. I was used to interviewing people but had to adapt my approach for these.

Through open discussions and mutual respect for each other's expertise, the ARG iteratively shaped the project concept, resulting in a rich and inclusive approach to storytelling. This process produced a website that reflected the richness of our combined insights and experiences and our inter‐organisational way of working:

What impressed me the most was the profound mutual respect for each other's expertise and individual competencies within the Action Research Group (ARG) we established. Our work within the ARG can be interpreted as the formation of a team with collective competencies aimed at narrating the story of the vaccination programme to the wider public and creating a public resource. (Creative/Arts Industries, Middlesex University, Cycle 2)

The widespread use of video meetings enabled inter‐organisational collaboration during the vaccination programme as well as during the ARG meetings. Previously groups of people across NCL would have had to physically travel to another organisation for a discussion, taking lots of time out of their working day. As one ARG member described it, “*my experience pre pandemic was that travelling to other hospitals was infrequent and usually only between two providers. Now, it's really easy for individuals across multiple organisations to jump on a 30 min teams call to collaborate*.”

By recording and analysing the testimonies of professionals and volunteers, this project managed to capture the processes that were engendered due to the constraints brought about by the pandemic and lockdown. This research showcases the new ways of working within a specified group of professionals, breaking silos and engaging in interprofessional collaboration. The process followed in our ARG was also shaped and enabled by the affordances brought about by the establishment of video conferencing for team meetings. This is also echoed in the way we worked together in the ARG; our bi‐weekly online video conferencing meetings and the recording of these created a vast wealth of content for us to draw from. It allowed for the automatic transcriptions of meetings, making analysis easier.

### 6.2 Theme 2 – Working Collaboratively in New Ways

The participants in cycles two and three included people from the NHS, academia, volunteers from a number of different occupations, external partners from local businesses, voluntary agencies and local councils (see Table 2); and within the NHS, professional disciplines were represented from HCPs, management, administration, finance and estates. These participants described the new ways of working which emerged through the vaccination programme which in turn, illustrated the inter‐organisational collaboration the programme developed. This quote by a communications manager in an NHS trust illustrates inter‐organisational working across a number of organisations within NCL to promote the vaccination programme:

we supported NHS colleagues in promoting pop‐up clinics for different groups at community venues and faith settings in the borough, e.g. during Ramadam, we ran late night pop‐up clinics for Muslims who were fasting during the day. We worked with Imams in the local mosques to develop communications for Muslims in the borough. (Cycle 2)

**TABLE 2:** ARG cycles participants' background.

Action research group cycles participant's disciplinary backgrounds	Cycle 1 number of ARG participants	Cycle 2 number of interview participants	Cycle 3 number of content contributors
Academic, arts and creative industries	1		
Academic, nursing/health (including trainer)	3		
Administrator		2	7
Commissioner			10
Communications		1	4
Doctor			1
Estates		2	4
External partner/local businesses, local councils			14
Finance		1	2
NHS communications team	1		
NHS Integrated Care Board (ICB^a^)	1		
NHS nursing	3		
NHS secondary care COVID‐19 vaccination lead	1		
NHS workforce	1		14
Nurse (including trainer)		3	22
Operational manager			17
Operations (with clinical background)		2	
Pharmacy		1	9
Training			7
Vaccinator			11
Volunteer			4
149 participants in total:	11	12	126

^a^ A statutory NHS organisation responsible for developing a plan for meeting the health needs of the population, managing the NHS budget and arranging for the provision of health services in the ICS area.

The vaccination teams were transient and diverse, brought together with the purpose of a mass vaccination campaign in NCL. These teams brought together individuals who created new ways of working which demonstrated inter‐organisational collaboration.

Huge privilege to take time out of normal role running the vaccination centre, cooperate between the NHS, XXX [local business venue], volunteers. Proud of level of collaboration between NHS and XXX [local business venue]. (Volunteer, Cycle 2)

Working in new teams in a national emergence with ‘*no blueprint’*, staff from across the NHS and partner organisations were deployed at short notice to set up the vaccination programme which was

Challenging…need for agility and flexibility in thinking and working differently – there seemed to be no answer but we had to find one…. there wasn't a blue print for this work, no guidance. (Estates, Cycle 2)

Working as a nurse in acute care for 20 years, working as a nurse on this outside the hospital with councils, public, volunteers, it was special, lovely. (Nurse, Cycle 3)

Vaccination work provided opportunities for people experiencing COVID job loss such as aircraft crew, hospitality sector workers who brought skills from one organisation to the new organisation.

It's given me opportunity, a job, being part of something extraordinary. So important to feel part of that and do[one's] bit for the community. (Volunteer, Cycle 2)

Captured on our website are audio clips of the creative team working, stories of rapid change; ‘*rapid upskilling’* and deployment of non‐healthcare workers into clinical roles. These new ways of working are described as ‘*breaking the mould*’. There are stories of personal transformation as well as accounts of new collaborations between population health providers, public health in local authorities and schools.

I'm most proud of were around increasing uptake amongst elderly ethnic communities. To ensure elderly people had protection. It was all through partnerships locally. Bus was good example of partnership working. Mosques, deprived parts of the borough. (Director PH local authority, Cycle 3)

There are accounts of collective action on health inequality – the vaccine outreach bus was cited many times in this respect – and of partnerships across the universities, NHS and Integrated Care System.

We‘ve got a really diverse population, so knowledge of the locality and population (which we had) was important. Engaging other group and faith leaders, monitoring uptake in different communities. We procured a vaccine bus and worked with local NHS providers. That was really successful. (Director Public Health local authority, Cycle 3)

For many participants, this work was life‐changing; for others, the first time they had worked in or with the NHS. Examples include a vaccination site lead who had worked prior to the pandemic on NHS trust communications at the strategic level, and during the vaccination programme, developed expertise in on‐site communications through contact with staff and the public coming for vaccines:

I'm most proud of working with my team who…. starting on something completely different …it was incredible to see them come together. (Site lead, business and communications background, Cycle 3)

For HCPs, particularly the trainers who were involved in leading the vaccination programme, the need to work inter‐organisationally was challenging as they were training members of the general public with no healthcare experience or knowledge to vaccinate. After decades of working as NHS university trainers, ‘silo’ organisational working and ‘silo’ professional teaching, trainers had to adapt to new ways of working; to train vaccinators for safe working in new organisations. This inter‐organisational collaboration produced a strong sense of risk and was mentioned several times in one video interview. Their anxiety was allayed, *a*nd as this nurse said:

we'd see them out in practice when we really needed the vaccine. I was working in ICU and seeing really sick patients and seeing bar staff, air staff, no experience, seeing them delivering vaccines safely was wonderful. Seeing people across different walks of life come together, give each other hope. (ARG member/nurse, Cycle 2)

This process of training staff who had been trained in non‐health occupations as vaccinators led to a powerful insight:

[People who] had never set foot in hospital before, musicians, dancers, financiers, cabin crew, people who do things involving muscle memory are the best vaccinators. (ARG/Nurse trainer, Cycle 2)

### 6.3 Theme 3 – Working Outside Professional, Occupational and Organisational Silos

The new ways of working in the vaccination programme involved new collaborations across professional, occupational and organisational boundaries. As discussed above in relation to vaccinator training, the professional tendency to draw on ‘knowledge silos’ means that professionals find it challenging to work collaboratively. Working collaboratively is a challenge to a silo mentality because it requires sharing knowledge and trusting others even though their worldview may be different from yours (as described by the ARG members above). This produced challenges that needed to be negotiated to make the vaccination programme succeed:

So many moving parts, having to work with people who had their issues, needed to tick boxes from their side and we were just ‘let's get it done’. (Senior nurse, Cycle 3)

The need to work outside the ‘normal’ was apparent to volunteers and experienced NHS managers alike:

I walked into something which had no structure – thinking on feet – totally new world working in NHS – that was new. Invigorating, this came at right time – loved it. (NHS manager, Cycle 3)

Problem solving on the run – if you can't solve it, work around it. Eyes and ears everywhere. (Volunteer, Cycle 3)

Being aware of one's knowledge silo enabled this senior nurse to accept a different point of view (related to risk) and use this trust in the other's good intentions to find a way forward despite the difference of views (or in this case, risk):

Working with people with different ways of looking at the world – people with more understanding of risk than me ‐ that was a challenge for me having people say hang on a minute….you'd be on calls with GPs in primary care who were worried. For me, I'd be thinking ‘just get it [vaccine] in. Just crack on’ and that was a learning [point] for me. To manage people's anxieties because I was used to managing such risk everyday. (ARG member/nurse, Cycle 2)

Working outside professional and organisational silos became imperative when it was recognised that the vaccination programme uptake was low amongst ‘difficult to reach’ groups. The vaccination team began to work in partnership with the local community to recruit vaccinators. One particularly effective strategy at this stage was to go out to local communities to increase vaccination uptake using the vaccination bus:

We worked on communications to reach the homeless and drug addicts. Focusing on high footfall venues proved successful. Our bus was ideal for this as in these high footfall areas our bus was highly visible. We developed partnerships between ourselves, NHS and local primary care centres. (Communications NHS, Cycle 3)

And working in ways that could be seen as unprofessional, to push the uptake of vaccinations up:

Aim was to offer vaccine to as many people as possible – so we turned the centre into a Christmas themed visit with free (donated mince pies) and staff dressed in Christmas jumpers, staff went out into street to pull people in. To appeal to people. (ARG/Nurse, Cycle 2)

As one vaccination centre manager said of working differently:

to set up the Bidborough House Art Project, to bring together local schools to provide art work thoughts, feelings and experiences about COVID. We filled the clinic with art… bright colourful space to welcome children. I learnt that in challenging times, it can bring out the best in people. There was so much kindness, teamwork and laughter. I am proud to have made a difference. (Deputy Ops lead, Bidborough House Art Project, Cycle 3)

It appeared to the research team that inter‐organisational collaboration was possible because of the ability of those engaged in the vaccination programme to work with a systems model rather than a professional model.

we can achieve everything if we just work together. (PH local authority, Cycle 3y)

Systems working was evident to volunteers:

As an American, to see socialised medicine from the inside has been fantastic. It's one of the reasons I stay in UK. It's an outstanding system. (Volunteer, Cycle 2)

Despite the vaccination programme being hugely demanding (as described in this paper), those involved were able to think about how they worked together as a local system:

You were […] aware of local work, collective work. (ARG/trainer, Cycle 2)

And as a wider system:

huge ambition and commitment….across health and social dynamic working programme…workforce to deliver safe vaccination programme….mobilise vaccination centres, as well as outreach, care homes and GP practice… challenge in deployment. (Regional NHSE, Cycle 3)

Although for a local business leader, entry into the NHS system was not always immediately obvious when they approached the NHS to see if a possible venue was suitable as a mass vaccination centre, the inter‐organisational collaboration between local councils and the NHS did work:

hardest thing was making contact with the right people in the NHS. Through contacts with local council hooked me up with senior NHS people. (Local business leader, Cycle 3)

There was a strong sense that the vaccination programme and the new ways it had engendered, of planning for the future after the pandemic as well as a way out of the pandemic at a time of national need. Cycles 2 and 3 data showed thinking and planning for the future and in particular, for the future workforce. The research team were repeatedly told by the NHS ARG members that out of the 3000 vaccinators trained during the programme, 218 have now got permanent NHS jobs, with 400 individuals in 2024 choosing to continue to work in the NHS on a temporary basis:

proud to give opportunity to people to change their lives. (ARG/NHS workforce, Cycle 2)

employing local people who'd never worked for NHS ‘local population engaging new generation in NHS in a national project. (ARG/nurse, Cycle 2)

We argue that systems thinking enabled staff on the vaccination programme to collaborate inter‐organisationally. This was described by NHS staff:

One giant improvisation…military project….challenging myself, managing conflicts in large teams. There's nothing in the book to help you, no‐one standing behind to step in…. you do your best. (Operations, Cycle 2)

And those in outside organisations:

Pretty much overnight and with very little resources. We had to increase the vaccine uptake in our borough. What we can be generally proud of is that we can achieve everything if we just work together. (Council Public Health Worker, Cycle 3)

oversight and coordination alongside NHS who were delivering the vaccination programme. (Director PH local authority, Cycle 3)

## 7 Discussion

Our findings illustrate how the strong inter‐organisational collaboration established by the vaccination team in the COVID‐19 vaccination programme shaped our inter‐organisational collaboration in this action research project, and ultimately, produced the artefact. The artefact is a witness to the inter‐organisational collaboration built by volunteers, HCPs, NHS managers and many others during the pandemic. During the vaccination programme and this action research study in the production of the artefact, people worked across disciplines and organisational boundaries, valuing each other's differences and working to incorporate each discipline in the shared work. They bridged professional, social, physical and task‐related gaps, negotiated overlaps in roles and tasks, and created spaces to communicate about interprofessional and inter‐organisational collaboration. Building on this experience amongst members of the vaccination programme team, the ARG were able to conduct a complex, multisite, action research study with 6 cycles of action/research. The artefact and three themes show how participants in cycles 2 and 3 described inter‐organisational working. In addition, the findings show how the vaccination team worked with volunteers, voluntary agencies, local councils and businesses, to create inter‐organisational collaboration and deliver a novel vaccination programme and then produce an artefact that reflected this inter‐organisational collaboration.

Collaboration is a mutually beneficial and well‐defined relationship entered into by two or more organisations to achieve common goals (Mattessich and Monsey 1992, 7). In the examples of inter‐organisational collaboration described in this paper, in total across the mass vaccination sites, there were 12 hospital trusts, six sites, five local authorities, NCL ICB, Middlesex University, UCL Partners, NHSE, volunteers such as the Samaritans, St John's, Age UK. In the ARG, there were three organisations working together. In the vaccination programme, the need and the goals arose in the national effort to survive a national emergence, the uncontained spread of COVID 19 and associated morbidity and mortality rates. Cycles 2 and 3 participants frequently referred to a national emergency. The collaboration that emerged out of this national emergency reflects what Green and Johnson (2015) describes as collaboration, namely, putting the community or client first, the organisation second and oneself last. Our findings show how professional silos were left behind and the vaccination team developed inter‐organisational collaboration to design and deliver a successful vaccination programme. In the inter‐organisational collaboration described in this paper, the vaccination programme team and the volunteers developed a commitment to defining mutual relationships and goals, a jointly developed structure and shared responsibility, mutual authority and accountability for success and shared resources. Rewards (which Mattessich and Monsey 1992, 7 argue are shared in collaboration) were not so much shared but there was a willingness to fund in different ways across partners. An example would be the offers from local businesses to provide space for mass vaccination centres; and for local schools to provide artwork.

As Yorke, Smith, and Mostrom (2022) describe, during the pandemic interprofessional collaboration promoted safety for patients and staff despite the chaos and uncertainty that changing roles and responsibilities in newly formed teams entailed. Our findings show that HCPs in the vaccination programme found it challenging to abandon professional silos when training vaccinators. But they did it because they realised the need for it and ultimately, learned that the training system they put in place was safe and as a result and developed new knowledge for the future.

D'Amour et al. (2008) and San Martín‐Rodríguez et al. (2005) suggest that for collaboration to occur adequate organisational arrangements are important. These include: clear common rules and suitable information structures as well as time, space and resources enabling professionals to get to know each other and to discuss issues that arise. D'Amour et al. (2008) and Nancarrow et al. (2013) argue that an open and receptive professional culture and a willingness to cooperate and communicate openly are also important. Such conditions necessary for collaboration are framed as a challenge for healthcare managers to promote and facilitate (Valentijn, Schepman, Opheij, and Bruijnzeels, 2013). Our findings show that whilst there were challenges and difficulties, inter‐organisational structures in the vaccination programme were established, were utilised in this action research study and continue to sustain work around vaccination and other health promotion programmes in NCL. Our findings would appear to support Green & Johnson's argument that professional silos are less constraining, more flexible to adaptation and national health burdens are being shared (2015). It may be that this is because professional silos appeared less important in a national emergency; that HCPs and their collaborators in NCL are working to sustain the inter‐organisational collaborative partnerships that were built during the pandemic and vaccination programme.

The paper documents the process put in place for interprofessional collaboration where professionals from different sectors came together to enact significant initiatives. Looking at the way NCL members united in the face of adversity, they organised a mass vaccination programme with little prior warning and no blueprint. Similarly, the ARG serves as an example of interprofessional collaboration, where professionals worked outside their traditional silos to produce an artefact for the public and commemorate the herculean efforts of staff and volunteers involved in the NCL Covid Vaccination programme.

This paper highlights two examples of working groups, out of the many that must have been formed during and after the pandemic, to showcase the new ways of working within a professional and highly skilled group of individuals – each with an already strongly established set of competencies. In the ARG, most members were in high positions and leadership roles, showcasing adaptability and leadership skills that were transferred and transformed to meet the aims of the research group.

## 8 Conclusions

The ways in which different sectors of health, social care, volunteers, voluntary agencies, local businesses and councils worked together appear to showcase the NHS at its best: flexible, innovative and patient‐centred. This way of working, which was developed during the vaccination programme in NCL, was then built on in the action research project described in this paper. It is reflected in the artefact (https://storiesbehindthescreens.net).

In this paper, we have shown how inter‐organisational collaboration amongst HCPs, their partners and researchers can help build informational networks, encourage different ways of thinking and stimulate new solutions to old problems (Green and Johnson 2015). We have shown that trust is an important factor for collaboration in practice as well as research although breaking professional barriers to achieve collaboration can be challenging (Green and Johnson 2015). The key challenge was navigating different workplace cultures. The academic approach of frequently re‐visiting ideas to strengthen them could feel frustrating for NHS colleagues who wanted decisions taken quickly – they had to learn some patience and trust the expertise of academic partners.

Considering some of the limitations of this research, it must be noted that our study focuses on education and health institutions, not the commercial sector. Academia, health and social care are sectors that require empathy and compassion in their daily operations. The ARG and NCL teams were comprised of members who care deeply about these values.

## Author Contributions

Helen T. Allan: Conceptualisation, Investigation, Formal analysis, Writing – original draft, Writing – review and editing. Sophia Drakopoulou: Conceptualisation, Evaluation design, Investigation, Formal analysis, Writing – review and editing. Miranda Willis: Evaluation design, Writing – review and editing. Michael Traynor: Evaluation design, Ethics application, Investigation, Formal analysis. Deborah Scott, Fiona Suthers, Karen Colfer, and Dan Levene: Evaluation design.

## Ethics Statement

The relevant fieldwork permissions were obtained and ethics approval received from Middlesex University, Arts & Creative Industries Ethics Committee in July 2022 and January 2023. Committee application number: 21632. All data utilised in the submitted manuscript have been lawfully acquired in accordance with The Nagoya Protocol on Access to Genetic Resources and the Fair and Equitable Sharing of Benefits Arising from Their Utilisation to the Convention on Biological Diversity.

## Conflicts of Interest

The authors declare no conflicts of interest.

## Peer Review

The peer review history for this article is available at https://www.webofscience.com/api/gateway/wos/peer‐review/10.1111/jan.16775.

## Data Availability

The data that support the findings of this study are available on request from the corresponding author. The data are not publicly available due to privacy or ethical restrictions.
